# Characterization of polyploid wheat genomic diversity using a high-density 90 000 single nucleotide polymorphism array

**DOI:** 10.1111/pbi.12183

**Published:** 2014-03-20

**Authors:** Shichen Wang, Debbie Wong, Kerrie Forrest, Alexandra Allen, Shiaoman Chao, Bevan E Huang, Marco Maccaferri, Silvio Salvi, Sara G Milner, Luigi Cattivelli, Anna M Mastrangelo, Alex Whan, Stuart Stephen, Gary Barker, Ralf Wieseke, Joerg Plieske, International Wheat Genome Sequencing Consortium, Morten Lillemo, Diane Mather, Rudi Appels, Rudy Dolferus, Gina Brown-Guedira, Abraham Korol, Alina R Akhunova, Catherine Feuillet, Jerome Salse, Michele Morgante, Curtis Pozniak, Ming-Cheng Luo, Jan Dvorak, Matthew Morell, Jorge Dubcovsky, Martin Ganal, Roberto Tuberosa, Cindy Lawley, Ivan Mikoulitch, Colin Cavanagh, Keith J Edwards, Matthew Hayden, Eduard Akhunov

**Affiliations:** 1 Department of Plant Pathology, Kansas State UniversityManhattan, KS, USA; 2 Department of Environment and Primary Industry, AgriBioSciences, La Trobe R&D ParkBundoora, Vic., Australia; 3 School of Biological Sciences, University of BristolBristol, UK; 4 US Department of Agriculture–Agricultural Research Service Biosciences Research LaboratoryFargo, ND, USA; 5 Commonwealth Scientific and Industrial Research Organization, Computational Informatics and Food Futures National Research FlagshipDutton Park, Qld, Australia; 6 Department of Agricultural Sciences, University of BolognaBologna, Italy; 7 Consiglio per la Ricerca e la sperimentazione in Agricoltura, Genomics Research CentreFiorenzuola d'arda, Italy; 8 Consiglio per la Ricerca e la sperimentazione in Agricoltura, Cereal Research CentreFoggia, Italy; 9 Commonwealth Scientific and Industrial Research Organization, Plant Industry and Food Futures National Research FlagshipCanberra, ACT, Australia; 10 TraitGenetics GmbHGatersleben, Germany; 11 IWGSCBethesda, MD, USA; 12 Department of Plant Sciences, Norwegian University of Life SciencesÅs, Norway; 13 Waite Research Institute, School of Agriculture, Food and Wine, University of AdelaideUrrbrae, SA, Australia; 14 Murdoch UniversityMurdoch, WA, Australia; 15 US Department of Agriculture–Agricultural Research Service Eastern Regional Small Grains Genotyping LaboratoryRaleigh, NC, USA; 16 Department of Evolutionary and Environmental Biology and Institute of Evolution, University of HaifaMount Carmel, Haifa, Israel; 17 K-State Integrated Genomics Facility, Kansas State UniversityManhattan, KS, USA; 18 INRA – Université Blaise Pascal, UMR 1095, Genetics Diversity and Ecophysiology of CerealsClermont-Ferrand, France; 19 Department of Crop and Environmental Sciences, University of Udine, Via delle ScienzeUdine, Italy; 20 Crop Development Centre and Department of Plant Sciences, University of SaskatchewanSaskatoon, SK, Canada; 21 Department of Plant Sciences, University of CaliforniaDavis, CA, USA; 22 Howard Hughes Medical InstituteChevy Chase, MD, USA; 23 Illumina Inc.Hayward, CA, USA

**Keywords:** single nucleotide polymorphism, polyploid wheat, wheat iSelect array, genotyping, high-density map, genetic diversity

## Abstract

High-density single nucleotide polymorphism (SNP) genotyping arrays are a powerful tool for studying genomic patterns of diversity, inferring ancestral relationships between individuals in populations and studying marker–trait associations in mapping experiments. We developed a genotyping array including about 90 000 gene-associated SNPs and used it to characterize genetic variation in allohexaploid and allotetraploid wheat populations. The array includes a significant fraction of common genome-wide distributed SNPs that are represented in populations of diverse geographical origin. We used density-based spatial clustering algorithms to enable high-throughput genotype calling in complex data sets obtained for polyploid wheat. We show that these model-free clustering algorithms provide accurate genotype calling in the presence of multiple clusters including clusters with low signal intensity resulting from significant sequence divergence at the target SNP site or gene deletions. Assays that detect low-intensity clusters can provide insight into the distribution of presence–absence variation (PAV) in wheat populations. A total of 46 977 SNPs from the wheat 90K array were genetically mapped using a combination of eight mapping populations. The developed array and cluster identification algorithms provide an opportunity to infer detailed haplotype structure in polyploid wheat and will serve as an invaluable resource for diversity studies and investigating the genetic basis of trait variation in wheat.

## Introduction

High-density single nucleotide polymorphism (SNP) data are widely used to detect marker–trait associations in quantitative trait locus (QTL) mapping experiments and genome-wide association studies (GWAS) (Cook *et al.*, 2012; Jia *et al.*, 2013; Tian *et al.*, 2011; Zhao *et al.*, 2011). Advances in next-generation sequencing have significantly facilitated the discovery of SNPs by whole genome (Berkman *et al.*, 2012; Chia *et al.*, 2012; Xu *et al.*, 2012), transcriptome (Allen *et al.*, 2011; Cavanagh *et al.*, 2013; Oliver *et al.*, 2013) or reduced-representation sequencing in diverse populations of individuals (Elshire *et al.*, 2011; Poland *et al.*, 2012; Saintenac *et al.*, 2011, 2013; Van Poecke *et al.*, 2013). Sets of informative SNPs selected based on their distribution across the genome, minor allele frequency (MAF) and intervariant linkage disequilibrium (LD), have been used to design high-density genotyping assays based on various technological principles (Cavanagh *et al.*, 2013; Ganal *et al.*, 2011; Kim *et al.*, 2007; Song *et al.*, 2013). While SNP arrays can be prone to ascertainment bias caused by preselection of SNPs in populations of limited size (Albrechtsen *et al.*, 2010), reduced computational requirements for downstream data processing, high call frequency, low error rate and ease of use make SNP-based platforms an attractive genotyping tool.

High-density SNP arrays have been developed for a number of economically important crops and animals (Ganal *et al.*, 2011; Sim *et al.*, 2012; Song *et al.*, 2013; Wiedmann *et al.*, 2008; Zhao *et al.*, 2011) and successfully used for genetic studies. The GWAS of 413 diverse rice accessions using a 44K SNP genotyping chip identified dozens of alleles controlling 34 morphological, developmental and agronomic traits (Zhao *et al.*, 2011). The 50K maize SNP chip has been used to study the genetic control of maize kernel composition in a nested association mapping panel (Cook *et al.*, 2012) and identify signatures of wild relative allele introgressions in the maize genome (Hufford *et al.*, 2012). The recently developed 9K SNP wheat chip was used to detect genomic regions targeted by breeding and improvement selection in wheat (Cavanagh *et al.*, 2013).

The allotetraploid and allohexaploid genomes of durum (*Triticum turgidum* subsp. *durum* (Desf.) Husnot*)* and bread wheat (*Triticum aestivum* L.), respectively, pose a significant challenge for the analysis of genotyping data generated using most SNP genotyping platforms (Akhunov *et al.*, 2009). The ratio of allelic variants observed in polyploids often deviates from the ratio observed in diploid organisms, resulting in genotype cluster plots (plots of the fluorescence intensities of the A and B alleles) that are difficult to analyse using conventional genotype calling software. In the polyploid wheat genome, this problem is further complicated by the presence of paralogous loci and secondary SNPs that interfere with genotyping oligonucleotide annealing (Akhunov *et al.*, 2009). While there have been attempts to develop cluster identification algorithms for polyploid genotyping data (Serang *et al.*, 2012), genotype calling in allopolyploid wheat still remains a significant challenge. In our previous study (Cavanagh *et al.*, 2013), we applied the default algorithm implemented in Genome Studio (Illumina) followed by extensive manual data curation. This approach resulted in high-quality genotype calls for many assays, but not for those that generated multiple clusters, closely spaced clusters or clusters with low fluorescence signal intensity. Further development of genotype calling procedures for polyploid species was required to accelerate the analysis of these complex data sets.

Here, we present the development of a wheat SNP iSelect array comprising of approximately 90 000 gene-associated SNPs that provides dense coverage of the wheat genome. To analyse the complex genotyping data generated for polyploid wheat, we applied two complementary model-free density-based clustering algorithms: OPTICS and DBSCAN (Ankerst *et al.*, 1999; Ester *et al.*, 1996). We demonstrate the utility of the developed array and genotype calling algorithms to reliably detect SNPs across worldwide wheat populations including hexaploid and tetraploid cultivars and landraces. A total of 46 977 SNP markers were genetically mapped using eight mapping populations, creating a resource for diversity studies and high-resolution dissection of complex traits in wheat.

## Results

### Variant discovery

For hexaploid wheat, more than 526 million quality-filtered RNA-seq reads (∼73 Gbp) were generated for 19 bread wheat accessions (Table S1). On average, 77% of reads from each accession were mapped to the reference transcripts (RTs). After quality filtering, 67 686 variants were discovered of which 72% were transitions and 28% were transversions. Among the 39 110 SNPs located in the protein-coding region, 24 460 SNPs were synonymous and 14 650 SNPs were nonsynonymous. Re-sequencing of sites polymorphic between accessions Kukri and RAC875 validated about 73% of SNPs (53 of 73) (Table S2), a result comparable to other wheat studies in which SNP discovery was performed using next-generation sequencing (Allen *et al.*, 2011; Cavanagh *et al.*, 2013; Edwards *et al.*, 2012; Lai *et al.*, 2012).

For tetraploid wheat, 666 million quality-filtered RNA-seq reads (∼64 Gbp) were generated for 18 cultivars selected from a worldwide collection of durum wheat (Maccaferri *et al.*, 2011) (Table S3) and one accession of emmer wheat (*T. turgidum* subsp. *dicoccum* Shrank ex Schübler Thell). Reads were mapped to RTs assembled for cultivar Svevo from ∼66 million reads (Table S4) and used to identify a total of 52 646 variants. The frequencies of transitions and transversions, and synonymous and nonsynonymous mutations were similar to those observed for bread wheat.

For assay design, we used the sets of SNPs discovered in this study with those previously identified in hexaploid wheat (Allen *et al.*, 2011; Cavanagh *et al.*, 2013; Pont *et al.*, 2013) combined with a small set of SNPs discovered by amplicon sequencing in a set of 24 varieties (M.Ganal unpublished data). To this marker set, SNPs from the diploid ancestor of the wheat D genome *Aegilops tauschii* (Luo *et al.*, 2013) were added. A total of 91 829 SNPs (Table S5) were included in the genotyping array, of which 261 and 91 568 were Infinium I (two probes per SNP) and Infinium II (one probe per SNP) assays, respectively. Of the 91 829 SNPs included in the original assay design, 81 587 (89%) passed the assay design process and produced functional assays.

Analysis of 81 587 nucleotide sequences corresponding to the functional iSelect SNP detection probes against the contigs assembled in the chromosome survey sequencing (CSS) project (http://wheat-urgi.versailles.inra.fr/Seq-Repository) identified 517 587 hybridization sites in the wheat genome. The average number of hybridization sites per probe was 6.3 with the median of three, suggesting that probes mostly targeted low-copy sequences in the wheat genome (Appendix S1, Figure S1). Using transcriptome and whole-genome shotgun sequences available for nine wheat varieties from the discovery panel (AC Barrie, Alsen, Baxter, Chara, Pastor, Volcani, Westonia, Xiaoyan54 and Yitpi), 25 252 (31%) of the SNPs could be assigned to a specific locus (on the A, B or D genome) in the CSS assemblies based on the association of the intervarietal polymorphism with sequence variation that distinguished between the hybridization sites on the different genomes to which the SNP detection probes were predicted to hybridize (Table S6). Comparison of the chromosomal assignments for 4538 of these SNPs that were also present on the 9K wheat iSelect assay and which had been previously genetically mapped (Cavanagh *et al.*, 2013) revealed 93.1% accuracy for the *in silico* assignments. The remaining 56 335 SNPs, which did not show polymorphism among these nine accessions, were tentatively assigned to wheat chromosomes based on the best blastn hit (based on percentage identity) of the nucleotide sequence flanking the SNP against the CSS contigs. Comparison of the tentative chromosomal locations for these SNPs with evidence from genetic mapping (Cavanagh *et al.*, 2013) indicated 79.6% accuracy for such assignments.

By comparing the flanking sequences of 81 587 SNPs, 13 357, 13 548 and 12 870, orthologous genes were uniquely tagged in *Brachypodium*, rice and sorghum, respectively (Table S7), providing a resource for comparative analysis of wheat genome.

### SNP genotype calling in polyploid wheat

**Figure 1:**
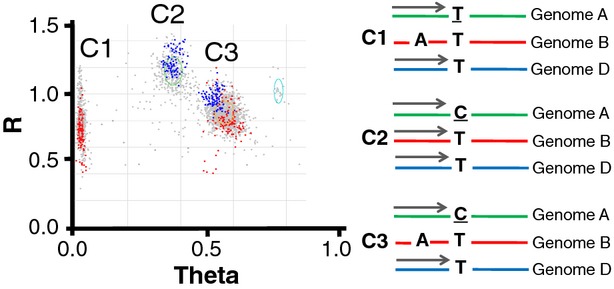
Assay IWB2818 shows multiple clusters in unrelated hexaploid wheat accessions, which can be tracked within bi-parental mapping populations as biallelic markers. The targeted [T/C] single nucleotide polymorphism (SNP) site is located in the A genome of hexaploid wheat. An SNP is located in the primer binding sequence of the B genome and results in the additional cluster (C3) on the genotyping plot due to failed/reduced hybridization for the assay oligonucleotide probe. Chara × Glenlea DH samples are shown in blue (situation C2/C3, polymorphism in Genome B). Westonia × Kauz DH samples are shown in red (situation C1/C3, polymorphism in Genome A). Diverse germplasm is shown in grey. Theta is the angle of deviation from pure T allele signal, where 0 represents pure T allele signal and 1 represents pure C allele signal; R is the intensity of hybridization signal. The graphical representation of genotypes in clusters C1, C2 and C3 is shown on the right side, where a grey arrow represents the Infinium probe.

As shown previously (Akhunov *et al.*, 2009; Cavanagh *et al.*, 2013), genotyping of polyploid wheat is complicated by the presence of duplicated (homoeologous and paralogous) genes. Due to low coding sequence divergence between homoeologous gene copies on different wheat genomes (2%–4%), and often between paralogous gene copies on the same genome, oligonucleotide probes can hybridize not only to the targeted locus, but also to its homoeologues and/or paralogues. As a consequence, the ratio of allele-specific fluorescent signals observed for an assay depends on the dosage of alternative SNP variants in the wheat genome. Increasing locus copy number reduces the ratio of allele-specific fluorescent signal, and the separation of SNP allele clusters (Figure 1). Wheat genotyping can be further complicated by the presence of mutations that modify oligonucleotide annealing sites located in one or more gene copies (Figure 1). This can result in assays that do not hybridize to all gene copies and show different cluster types.

**Figure 2:**
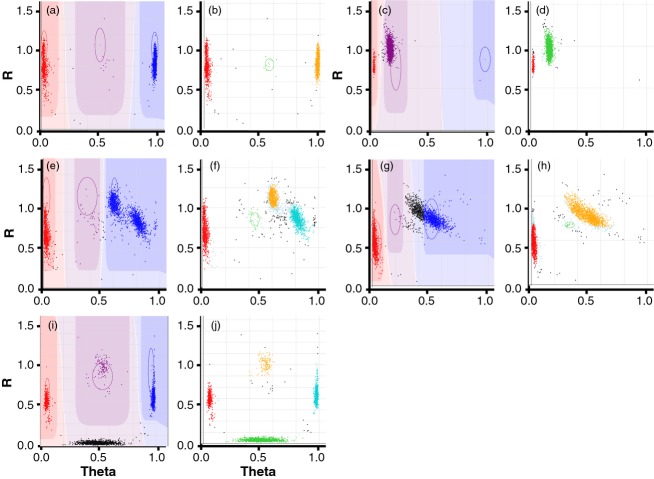
Examples of clustering obtained using diploid and polyploid versions of the GenomeStudio software, respectively: (a, b) assay IWB8846; (c, d) assay IWB63414; (e, f) assay IWB36584; (g, h) assay IWB15488; and (i, j) assay IWB54207.

We applied the standard diploid version of GenomeStudio (GS) software (Illumina) to call genotypes for the iSelect 90K SNP assay. For this purpose, a diverse worldwide panel of almost 2500 hexaploid accessions was assembled and used to develop a cluster file storing information about cluster positions on the genotyping plot. A total of 35 684 (44%) assays showed three distinct clusters corresponding to the AA, AB and BB genotypes expected for a biallelic SNP (Table S8): 20 785 had well-separated clusters that were correctly captured by the default algorithm (Figure 2a); 9960 had poor cluster separation, for which manual clustering was required and heterozygous genotypes could not be called (Figure 2c); and 4939 showed four clusters. Of the remaining assays, 25 199 (31%) were monomorphic (consistent with 73% Sanger-based validation rate) and 20 704 (25%) showed complex clustering patterns that could not be correctly captured even with manual curation (Figure 2e,g,i). Similar proportions of polymorphic and monomorphic sites were identified in the SNP discovery panel. Overall, 56 388 (69%) of the 81 587 functional iSelect bead chip assays visually revealed polymorphism among the unrelated wheat accessions, of which 35 684 (63% of 56 388) could be correctly clustered for genotype calling providing six times more markers than the previously developed 9K iSelect assay (Cavanagh *et al.*, 2013). In a diverse set of 55 tetraploid cultivars and landraces, 20 197 SNPs showed clustering corresponding to bi-allelic sites. A total of 36 037 biallelic SNPs segregated in the populations of both tetraploid and hexaploid wheat.

The shortcomings of the standard version of the GS software for analysing polyploid genotyping data are its inability to identify multiple (>3) clusters, its inability to call heterozygous genotypes when clusters are compressed due to the hybridization of assay probes to duplicated targets, and the requirement for time-consuming manual curation of assays incorrectly clustered by the default algorithm. To address these shortcomings, we used two model-free density-based cluster identification algorithms: DBSCAN (Ester *et al.*, 1996) and OPTICS (Ankerst *et al.*, 1999). Both algorithms can detect any number of clusters of arbitrary shape. They each require only two user-defined input parameters, ‘minimum number of points in cluster’ and ‘cluster distance’. The first parameter specifies how many data points need to be inside a circular cluster distance area to be able to form a cluster, while the second parameter defines the minimum separation distance between clusters for clusters not to merge. Together, these two parameters define the density of the cluster areas. The ‘minimum number of points in cluster’ parameter helps to minimize the merging of two or more clusters that are not fully separated. A modified OPTICS algorithm can identify a user-defined number of clusters. To increase speed for manual annotation, the polyploid version of GS was developed by Illumina that currently implements both of these algorithms.

Using these algorithms in combination with a cluster file developed using multiple bi-parental mapping populations, we identified clusters in genotyping data sets from unrelated wheat lines (Appendix S1, Figures S2–S4, Tables S9, S10). Among the 56 388 assays that exhibited visible polymorphism, 46 880 (83%) had more than a single cluster correctly captured. For the other 9508 assays, only one of the observed clusters was captured, indicating that one or more additional clusters on a genotyping plot were not present in any of the six mapping populations used for cluster file development. Only 1783 (4%) of the 48 663 assays revealing polymorphism in the six mapping populations were not present in the unrelated accessions. Inclusion of additional mapping populations at the cluster file development stage should increase the number polymorphisms that can be correctly called in diverse populations.

To confirm the accuracy of the clustering, we compared genotype calls produced by the diploid and polyploid versions of GS for biallelic assays with three clusters corresponding to the AA, AB and BB genotypes. The concordance between the two data sets was 99.6%, and the overall cluster assignment rate was 99% and 97% for the diploid and polyploid versions of GS, respectively. The differences in genotype and cluster assignment rates were primarily due to three factors: (i) low data density, especially for heterozygous genotypes that prevented cluster identification using DBSCAN and OPTICS. This was most notable for SNPs that likely had single-dose occurrence in the wheat genome and produced well-spaced clusters (Figure 2a,b); (ii) cluster compression (Figure 2c,d) and irregular cluster shape (Figure 2g,h) that prevented complete data capture by the default diploid algorithm; and (iii) application of the Confidence Score Limit in the polyploid version to exclude nonreliable data.

To assess the accuracy for near-automated genotype clustering in mapping populations (3-step procedure described in Appendix S1), we used the polyploid GS to identify polymorphisms in two doubled-haploid mapping populations. The majority (average 79%) of SNPs were detected in the first step (Table S11). The remaining SNPs were captured mostly in the second step, in which the rate of incorrectly clustered assays increased to an average of 5.9%. Visual inspection of 5000 randomly selected assays for which only a single cluster was detected revealed ∼5% rate for missed polymorphisms. Genotype calling of the same mapping populations using the cluster file developed for the diploid version of GS revealed substantially fewer polymorphic assays: 11 187 and 11 877 in the Chara × Glenlea and Young × AUS33414 populations, respectively.

#### Construction of genetic maps

Eight doubled-haploid mapping populations were used to order SNPs along wheat chromosomes. Genotype calling was performed using the polyploid version of GS. A total of 45 109 assays revealed polymorphism in the mapping populations (Tables S12 and S13). Of these assays, 44 345 could be mapped to one or more of 46 977 loci on specific wheat chromosomes. Of the remaining 764 polymorphic assays, 20 mapped to linkage groups that could not be unambiguously assigned to a wheat chromosome, and 744 were not linked with any other markers. Of the assays revealing polymorphism that could be mapped on wheat chromosomes 41 746 mapped to a single position, 2508 to two different positions, 69 to three positions and two to four positions. Consistent with previously observed levels of genetic diversity in the wheat genomes, the majority of mapped markers were located in the A (35%) and B (50%) genomes. Only 15% of markers mapped to the D genome (Table 1).

**Table 1:** Distribution of mapped SNP loci across the wheat genome

Chromosomes	Wheat genome			Total
	A	B	D	
1	2260	4020	1082	7362
2	2502	6456	1561	10 519
3	1975	2739	899	5613
4	2017	1513	320	3850
5	2672	3347	1120	7139
6	2369	2810	618	5797
7	2867	2526	1304	6697
Total	16 662	23 411	6904	46 977

SNP, single nucleotide polymorphism.

**Figure 3:**
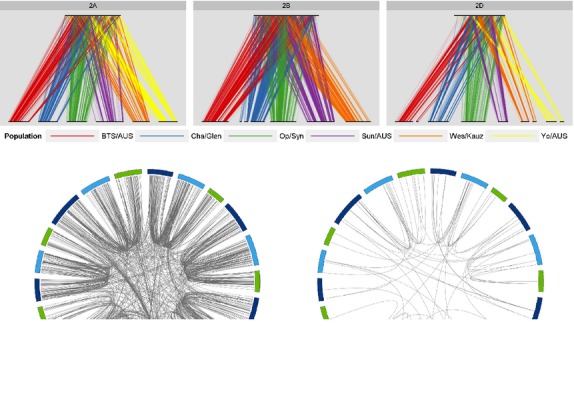
(a) Alignment of chromosome 2 consensus maps with genetic maps from individual bi-parental crosses. BTS/AUS = BT-Schomburgk × AUS33384, Cha/Glen = Chara × Glenlea, Op/Syn = W7984 × Opata M85, Sun/AUS = Sundor × AUS30604, Wes/Kauz = Westonia × Kauz, Yo/AUS = Young × AUS33414. Chromosome 2B from Yo/AUS was excluded from consensus map construction due to the presence of the alien *Sr36* introgression in cultivar Young, whose presence restricts recombination and complicates map construction. (b) Comparative analysis of the order of single nucleotide polymorphism (SNP) loci in the wheat genome based on SNPs showing segregation at two (left) and three (right) duplicated loci.

Six of the doubled-haploid mapping populations were used to construct a consensus SNP map containing 40 267 loci (Table S13). Comparison of the consensus map order with that obtained for individual populations showed high collinearity across chromosomes, confirming the high accuracy of genotype calling using the polyploid GS (Figure 3a). Comparative analysis of SNP order revealed by assays detecting segregation at nontarget SNPs (see below) showed the high level of gene order conservation between homoeologous chromosomes, as well as frequent gene duplications across chromosomes (Figure 3b). These assays provide insights into the structural organization of the wheat genome revealing new and previously characterized re-arrangements (Devos *et al.*, 1995).

#### Identification of nontarget SNPs and null alleles

The ability for the polyploid clustering algorithms to detect any number of clusters allowed for the capture of genotypic data for SNP assays that detected polymorphism at nontarget SNPs located on homoeologous chromosomes or duplicated paralogous targets on different chromosomes. Such assays showed more than the three expected clusters for a biallelic SNP when genotyped in unrelated germplasm but could be resolved as biallelic markers in segregating bi-parental mapping populations (Figure 1). A total of 25 643 assays detected multiple clusters in the population of unrelated hexaploid wheat accessions, representing 31% (25 643/81 857) of the entire content in the iSelect 90K bead chip array, and 46% (25 643/56 388) of all polymorphic assays. Using eight mapping populations, we were able to map polymorphisms revealed by 18 360 (72%) of these assays.

**Figure 4:**
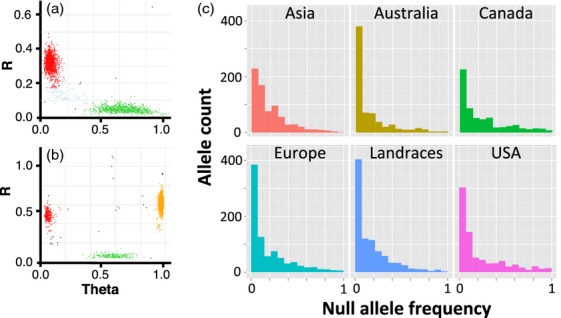
Examples of null alleles in the wheat genome. (a) Assay IWB17050 detecting a null allele; (b) Assay IWB12859 detects a co-dominant single nucleotide polymorphism locus that also shows the evidence of a null allele; (c) Frequency of nulls in the populations of different geographical origin.

The ability of the clustering algorithms implemented in the polyploid version of GS to detect clusters of any shape allowed for the identification of null alleles (clusters with low signal intensity) resulting from either the deletion of single-copy genes in the wheat genome or the divergence of genotyping probe annealing sites (Figure 4). A total of 1660 single-locus SNPs showed evidence for null alleles. We investigated the molecular basis of null allele origin by comparing the sequences of SNP probes detecting these alleles in wheat cultivar Chinese Spring with the genomic sequence of this cultivar. Based on the comparison of flanking sequences of 94 SNP assays detecting the null alleles in cultivar Chinese Spring, 46 assays did not have annealing sites in the genome. This result suggests that about 50% of null alleles result from gene deletions and remaining are the consequence of sequence divergence at the SNP probe annealing sites.

### Genetic variation assessment using the 90K wheat SNP assay

The 90K iSelect genotyping assay was tested by surveying SNP variation in a samples 550 hexaploid and 55 tetraploid wheat accessions including landraces and cultivars of different geographic origin from North America, Australia, Europe and Asia (Table S14). The number of biallelic polymorphic loci per population varied from 12 524 in Australian material to 21 110 in European material (Table 2). The level of genetic diversity in the cultivars was either comparable or higher than that of the population of landraces, possibly due to ascertainment bias in the SNP discovery panel, which comprised mainly of cultivars.

**Table 2:** SNP diversity summary assessed in the populations of wheat cultivars and landraces

Populations	Ploidy	Accessions	Mean heterozygosity	Number of polymorphic bi-allelic SNPs
Asia	6n	29	0.20	16 968
Australia	6n	182	0.24	12 524
Canada	6n	46	0.17	15 427
Europe	6n	71	0.18	21 110
USA	6n	95	0.15	17 013
Landraces	6n	127	0.20	17 984
Durum wheat	4n	55	0.07	20 197

SNP, single nucleotide polymorphism.

**Figure 5:**
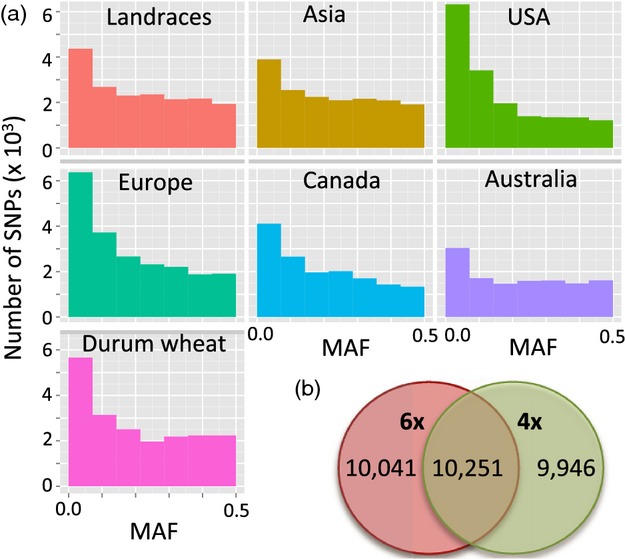
Single nucleotide polymorphism (SNP) distribution across populations. (a) Minor allele frequency across populations of different origin. (b) Shared and private SNPs between the analysed tetraploid and hexaploid wheat populations.

To ascertain the transferability of SNP markers across populations, we assessed the number of shared alleles and the degree of genetic differentiation (*F*_ST_) between the wheat populations (Table 3). The majority of polymorphic SNPs were shared among populations, suggesting that the targeting of SNPs with both alleles present in at least two individuals in the discovery panel enriched the array for common SNP variants. This observation is consistent with the prevalence of SNPs of intermediate to high MAF in the populations (Figure 5a). *F*_ST_ variation between the populations of different geographical origin is likely caused by the usage of different founders (Table 3) and/or by allele frequency divergence during the development of locally adapted populations. For example, broad usage of landraces in the breeding programmes of Asia could have resulted in low *F*_ST_ between landraces and Asian cultivars (Cavanagh *et al.*, 2013). Our analyses also confirm previous observations showing the high proportion of shared alleles between wheat cultivars as a whole and landraces (Cavanagh *et al.*, 2013), suggesting that the majority of alleles for wheat improvement were contributed by landraces.

**Table 3:** The number of SNP markers shared between populations (above diagonal) and the estimates of pairwise *F*_ST_ (below diagonal)*

	Landraces	Asia	USA	Europe	Canada	Australia
Landraces		15 823	14 770	16 312	14 772	8173
Asia	0.02		14 448	15 773	14 501	7842
USA	0.10	0.15		15 920	13 761	7908
Europe	0.11	0.11	0.18		14 867	8645
Canada	0.17	0.16	0.28	0.22		7442
Australia	0.26	0.26	0.32	0.31	0.31	

SNP, single nucleotide polymorphism.

* Weir and Cockerham's unbiased pairwise *F*_ST_.

The 90K assay included 4427 functional SNP assays discovered by re-sequencing two subspecies of *Ae. tauschii* (ssp. *tauschii* and ssp. *strangulata*) (You *et al.*, 2011). Of these SNPs, 2827 SNPs were bi-allelic in the panel used for training the clustering algorithms (Tables S5 and S8). As only one of the *Ae. tauschii* haplotypes was closely related to the wheat D genome (Wang *et al.*, 2013), we expected that the majority of these SNPs would be monomorphic in hexaploid wheat. Consistently, in a set of 550 hexaploid wheat lines (Table S14), only 796 of these SNPs (18%) were polymorphic. However, in mapping populations developed using synthetic wheats created by hybridizing tetraploid wheat with *Ae. tauschii*, the fraction of segregating SNPs was significantly higher. For example, of 1332 genetically mapped SNPs discovered in *Ae. tauschii*, 1219 were polymorphic only in the synthetic wheat mapping populations.

For a set of SNPs mapped to the A and B genomes, we assessed the proportion of shared alleles between tetraploid durum and hexaploid bread wheat populations. Of 30 238 biallelic SNPs in durum (pasta) and hexaploid wheat populations, 10 251 SNPs (34%) were shared, consistent with the previous observation (Dvorak *et al.*, 2006) that there was an extensive gene flow from the populations of tetraploid ancestors to hexaploid wheat (Figure 5b). Of 8906 variants discovered by sequencing the durum wheat transcriptome (Table S5), there were nearly two times more SNPs (3691) that were polymorphic in tetraploid than in hexaploid wheat (1777).

The extent of LD, the nonrandom association of alleles at different loci, was assessed in the populations of cultivars and landraces. Consistent with the effect of wheat improvement on LD (Cavanagh *et al.*, 2013), the rate of LD decay was higher in landraces than in cultivars (Figure S5). Likewise, our analysis confirmed previously observed genome-specific LD patterns in the wheat genomes (Chao *et al.*, 2010) with LD in the D genome decaying two to three times slower than in the A and B genomes.

## Discussion

We present the development of a resource for high-density genotyping of wheat using a custom iSelect bead array assaying 81 587 gene-associated SNPs. The utility of the iSelect assay for functional studies in wheat was maximized by anchoring the SNPs to CSS contigs with high (93%) accuracy for chromosome assignment, identifying orthologous genes in *Brachypodium*, rice and sorghum, and generating genetic maps containing 46 977 loci. The MAF of SNP alleles ranging from intermediate to high in the populations of different origin suggests high transferability of SNP markers. The value of the iSelect array for genetic studies and breeding of durum and bread wheat was enhanced by including SNPs discovered in diverse populations of tetraploid and hexaploid wheat. The inclusion of SNPs polymorphic in *Ae. tauschii* provides an opportunity to analyse variation in this wild species and to map introgressions of genetic material from this wild relative which has been extensively used as a source of alleles contributing to abiotic and biotic stress tolerance in wheat (Jones *et al.*, 2013; Periyannan *et al.*, 2013; Sohail *et al.*, 2011).

The model-free density-based clustering algorithms implemented in the polyploid version of GS provided a significant improvement for genotyping polyploid wheat. While the requirement to visually inspect each SNP remains, manual curation of incorrectly clustered SNPs is simplified by a modified OPTICS algorithm that allows automatic re-clustering of an assay for a user-defined number of clusters. The polyploid version of GS also has the ability to detect densely spaced clusters or clusters of arbitrary shape. One of the useful applications of OPTICS and DBSCAN algorithms was for chromosomal assignment of alleles for assays that revealed multiple clusters due to segregation at more than one duplicated locus. Assays revealing multiple clusters in unrelated wheat accessions tended to segregate as biallelic markers in bi-parental mapping populations. By tracking cluster positions for loci that segregated in the mapping populations, it was possible to establish the allelic relationship between the multiple clusters observed in unrelated wheat accessions. This strategy allowed us to establish the allelic relationship between clusters for 72% (18 360) of the 25 643 assays showing multiple clusters. This capability provides opportunities to better utilize assays that reveal segregation at more than one duplicated locus in genetic diversity studies, GWAS and for investigating structural variation in the wheat genome.

The clustering algorithm reliably detected clusters showing low signal intensity due to divergence of SNP assay probe hybridization sites or presence–absence variations (PAVs). The latter type of variation was shown can contribute to phenotype (Chia *et al.*, 2012; Springer *et al.*, 2009), and the resources developed here will provide an opportunity to investigate the impact of PAVs on trait variation in wheat.

Single nucleotide polymorphisms on the array were shown to be polymorphic across multiple populations of different geographical origin, suggesting that the array can be used as a genotyping platform in various wheat genetic studies. A high proportion of shared SNPs is likely the result of using common founders for developing regional populations and intercrossing of relatively few locally adapted cultivars in regional breeding programmes (Chao *et al.*, 2010). In spite of the significant fraction of SNPs shared among landraces and cultivars, we observed differentiation in allele frequency between regional populations and landraces. This allele frequency shift can be attributed to several factors, including disproportional usage of a limited number of founders in developing regional populations and enrichment of alleles associated with regional adaptation by local breeding programmes (Cavanagh *et al.*, 2013). This conclusion is consistent with the effect of wheat improvement on patterns of LD. The observed elevated correlation of alleles in wheat cultivars compared with that landraces is suggestive of a population bottleneck probably caused by the usage of limited number of landrace accessions in breeding.

In conclusion, the developed 90K array, genotype calling algorithms and high-density genetic maps provide a useful resource for analysing genome-wide variation in wheat. The high data quality and low proportion of missing genotypes provide an opportunity to create a high-resolution haplotype map of the wheat genome and build a framework for future analyses of genomic variation in mapping experiments and diversity studies. A haplotype map of wheat will serve as a resource for the extrapolation of data across diversity studies and imputation of missing genotypes in experiments using low-coverage sequencing as a genotyping tool. These developments will advance the field of wheat genetics and genomics and help in elucidating intricate relationships between phenotype and genotype.

## Experimental procedures

### Plant material

The distribution of the 90K SNPs across populations was assessed in the diverse panel of 726 accessions including tetraploid and hexaploid landraces (Table S14). A total of eight bi-parental doubled-haploid mapping populations were used to order SNPs along chromosomes: BT-Schomburgk × AUS33384 (CIGM92.1712), Young × AUS33414 (CIGM93.238), Chara × Glenlea, W7984 × Opata M85, Sundor × AUS30604, Westonia × Kauz, Avalon × Cadenza and Savannah × Rialto. Ditelosomic lines for Chinese Spring wheat (Kimber and Sears, 1968) were used to test the accuracy of clustering and assign the consensus genetic map linkage groups to wheat chromosomes. For cluster file development for hexaploid wheat, 2473 bread wheat lines comprising 1979 worldwide wheat accessions and 494 F_4_ progeny from a nested association mapping population were used. The F_4_ lines were included to provide a sufficient number of heterozygous individuals for the majority of SNPs to ensure correct clustering of the heterozygous SNP alleles. For cluster file development in durum wheat, diverse accessions from a worldwide durum panel, recombinant inbred lines from a four-way cross of (Neodur × Claudio) × (Colosseo × Rascon37/Tarro2/Rascon37), six F_1_ samples (Dylan × Normanno; Tiziana × Normanno; Dupri × Normanno; Achille × Normanno; Strongfield × Saragolla; Kofa × Claudio) and the corresponding nine F_1_ parental lines were used.

### SNP discovery

The RTs of tetraploid and hexaploid wheat were generated by assembling RNA-seq data generated using several next-generation sequencing platforms (Appendix S1). SNP discovery was performed in the transcriptomes of 19 accessions of hexaploid (Table S1) and 18 accessions of tetraploid (Table S3) wheat.

### Selection of SNPs for the genotyping assay design

For assay design, SNPs were filtered to remove those that (i) had sequences showing similarity to the repeats (*e*-value ≤1*e*−10) identified by comparing 100 bp SNP-flanking sequences with the GIRI (http://www.girinst.org/repbase/) and ITMI Triticeae Repeat Sequence databases (wheat.pw.usda.gov/ITMI/Repeats) and (ii) were located in close proximity (<50 bp) to the exon–intron junctions identified in the wheat genome assembly (Brenchley *et al.*, 2012). The selected SNPs were then submitted to the Illumina Assay Design Tool for design score calculation (www.illumina.com). A total of 91 829 SNPs were included into the assay design (Table S5).

Synonymous or nonsynonymous SNPs were annotated by comparing sequences with the nonredundant protein database at NCBI (https://www.ncbi.nlm.nih.gov/) using the blastx program with the e-value threshold of ≤1*e*^−10^. For functional annotation, RTs were translated into six reading frames and compared against the protein sequences (blastx *e*-value threshold ≤1*e*^−05^) predicted in the rice, sorghum, maize and barley genomes. The output of the blastx program was used for automated functional annotation using blast2GO (http://www.blast2go.de/).

### SNP genotype calling using the diploid version of Genome Studio (GS)

Single nucleotide polymorphism allele clustering and genotype calling for tetraploid and hexaploid wheat was performed with GS v2011.1 as described in Cavanagh *et al*. (2013)). In brief, the default clustering algorithm implemented in GS was first used to identify assays that produced three distinct clusters corresponding to the AA, AB and BB genotypes expected for biallelic SNPs. Manual curation was performed for assays that produced compressed SNP allele clusters that could not be discriminated by the default algorithm. The accuracy for SNP clustering was validated visually.

### SNP genotype calling in hexaploid wheat using the polyploid version of GS

Single nucleotide polymorphism clustering was performed with GS Polyploid Clustering v1.0 software using the three steps described in Appendix S1. In the first step, the density-based DBSCAN clustering algorithm (Cluster Distance = 0.07 and Minimum Number of Points in Cluster = 10) was used to identify assays producing one or more clusters. The DBSCAN does not have an *a priori* expectation for the number of clusters and can find arbitrarily shaped clusters (Ester *et al.*, 1996). The setting of the minimum number of points in a cluster to ten helped to minimize the merging of clusters into a single cluster when clusters were not well separated. The clustered SNPs were then filtered based on custom cluster number, call rate and MAF. In the second step, SNP assays for which only a single cluster was detected in the first step were re-clustered using the OPTICS (Ankerst *et al.*, 1999) clustering algorithm (Cluster Distance = 0.07, Minimum Number of Points in Cluster = 10 and Force Two Clusters option). This step allowed the identification of two clusters that were closely spaced due to the presence of duplicated copies of the SNP locus in the wheat genome. Similar to the first step, assays with two clusters were filtered based on cluster number. In the third step, assays for which satisfactory SNP clustering was not yet achieved were re-clustered using the DBSCAN algorithm with parameters Cluster Distance = 0.09 and Minimum Number of Points in Cluster = 10, followed by filtering based on custom cluster number, call rate and MAF. This step allowed for the identification of clusters that were too broad to be detected in the first DBSCAN. Finally, wheat accessions were assigned to a SNP cluster for each assay using a Confidence Score Limit of 0.8. A MAF of 0.35 was used to filter SNP clustering performed for genetic mapping populations, and a MAF of 0.05 was used to filter SNP clustering for unrelated wheat accessions. The accuracy for SNP clustering was visually checked, and incorrectly clustered SNPs were manually curated. Sample cluster assignments for each SNP assay were converted to genotype calls (Appendix S1, Figures S3 and S4).

### Data analyses

Basic summary statistics for each SNP (MAF, average heterozygosity and *F*_ST_) and LD were calculated using R package *genetics*. The linkage map was constructed using the MSTmap program (Wu *et al.*, 2008). Linkage groups were assigned to chromosome based on the best blastn hit from a comparison of SNP-flanking sequences with the CSS sequences. The program MergeMap (Wu *et al.*, 2011) was used to construct the consensus map using the previously described strategy (Cavanagh *et al.*, 2013).

## Supporting Information

Figure S1 Cumulative distribution of the number of putative hybridization sites for genotyping oligonucleotides.

Figure S2 Sequential addition of mapping populations during cluster file development in polyploid version of GenomeStudio.

Figure S3 Calling genotypes at the targeted SNP locus.

Figure S4 Calling sample genotypes for iSelect assays that detect multiple clusters in a population of unrelated wheat accessions.

Figure S5 LD decay in the populations of wheat cultivars and landraces.

Table S1 Summary of sequencing data generated for wheat transcriptome.

Table S2 SNP validation.

Table S3 Durum wheat genotypes used for SNP discovery.

Table S4 Summary of RNA-seq data generated for cultivar Svevo.

Table S5 Annotation of SNP loci.

Table S6 Assignment of SNPs to a specific locus in the wheat genome using CSS assemblies.

Table S7 Blast hits of SNP flanking sequences against CDS and protein sequences of *Brachypodium*, rice and sorghum.

Table S8 Annotation of clustering patterns observed for SNP assays.

Table S9 Proportion of iSelect 90K bead chip assays trained to capture polymorphisms.

Table S10 Theoretical expectations for segregation at a single-copy, duplicated and triplicated SNP locus.

Table S11 Numbers of assays that reveal polymorphism in the Chara × Glenlea and Young × AUS33414 mapping populations.

Table S12 Genetic linkage maps for bi-parental doubled-haploid mapping populations.

Table S13 Consensus SNP genetic linkage map for hexaploid wheat.

Table S14 Hexaploid and tetraploid wheat accessions used to assess the distribution of the 90K SNPs across populations.

Appendix S1 Methods (SNP discovery, cluster file development, map construction).
