# Supplementary material for: Characterization of polyploid wheat genomic diversity using a high-density 90 000 single nucleotide polymorphism array
Source: Plant Biotechnol J. 2014 Mar 20;12(6):787–96. doi: 10.1111/pbi.12183 (PMC4265271; doi:10.1111/pbi.12183)
Supplement: Figure S1 — Cumulative distribution of the number of putative hybridization sites for genotyping oligonucleotides. [file pbi0012-0787-SD1.pdf]

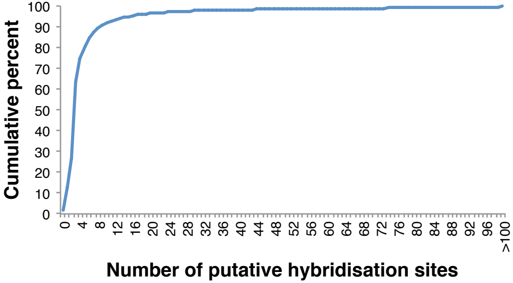

Figure S1. Cumulative distribution of the number of putative hybridization sites in the wheat genome for genotyping oligonucleotides.
