# Supplementary material for: Characterization of polyploid wheat genomic diversity using a high-density 90 000 single nucleotide polymorphism array
Source: Plant Biotechnol J. 2014 Mar 20;12(6):787–96. doi: 10.1111/pbi.12183 (PMC4265271; doi:10.1111/pbi.12183)
Supplement: Figure S2 — Sequential addition of mapping populations during cluster file development in polyploid version of GenomeStudio. [file pbi0012-0787-SD3.pdf]

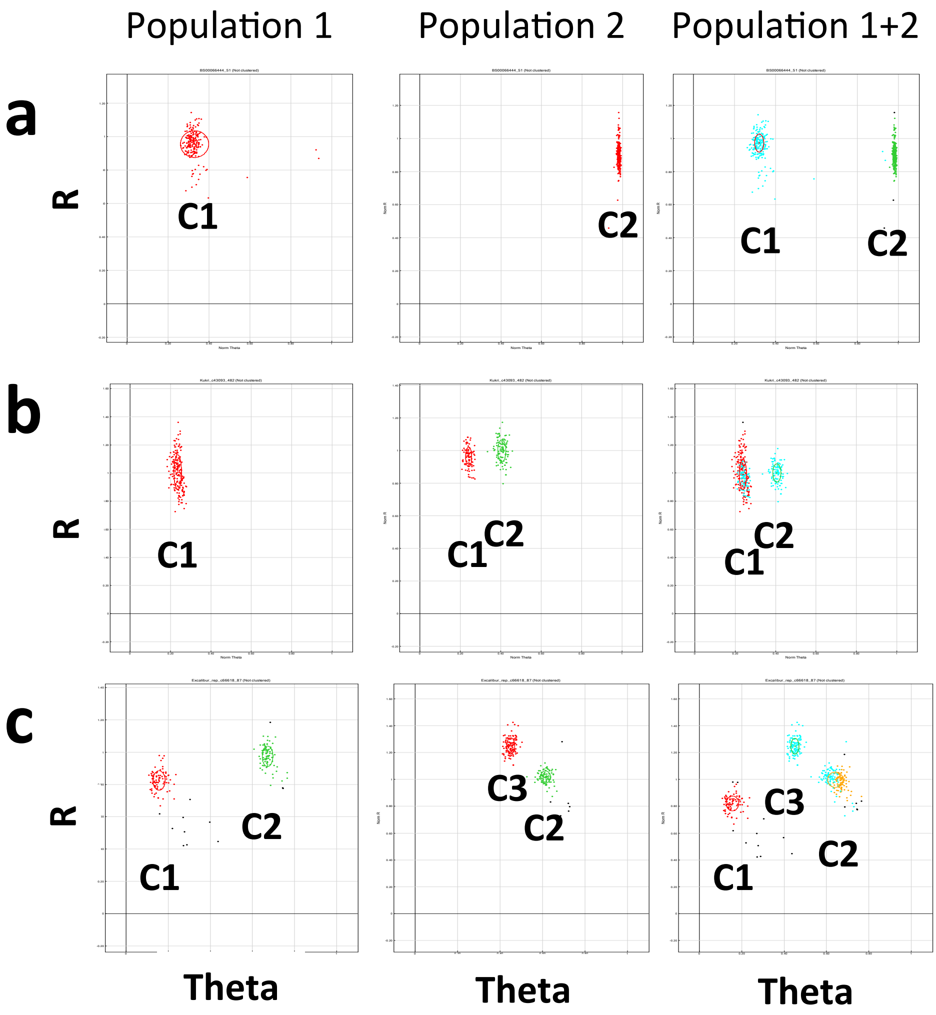

Figure S2. Sequential addition of mapping populations during cluster file development in polyploid version of GenomeStudio. (a) SNP is monomorphic in both mapping crosses but fixed for alternate alleles. Inclusion of both populations in the same Genome Studio project creates artificial polymorphism that allow correct clustering of both alleles; (b) SNP is monomorphic in one cross and polymorphic in the other. Inclusion of both populations in the same project results in correct capture of both alleles; and (c) SNP is polymorphic in both populations but segregation occurs at different duplicated loci. Inclusion of both populations in the same project allows correct clustering of all alleles.
