# Supplementary material for: Characterization of polyploid wheat genomic diversity using a high-density 90 000 single nucleotide polymorphism array
Source: Plant Biotechnol J. 2014 Mar 20;12(6):787–96. doi: 10.1111/pbi.12183 (PMC4265271; doi:10.1111/pbi.12183)
Supplement: Figure S3 — Calling genotypes at the targeted SNP locus. [file pbi0012-0787-SD5.pdf]

(a) IWB18 - SNP [A/G] mapped in chromosome 2B

Cluster #	C1	C2	C3
Cluster genotype	AA	AG	GG
Locus 1 (2B)	AA	AG	GG
Theta value	0.00	0.50	1.00

(b) IWB16 - SNP [T/C] mapped in chromosome 5B

Cluster #	C1	C2	C3
Cluster genotype	TT	TC	CC
Locus 1 (5B)	TT	TC	CC
Locus 2	CC	CC	CC
Theta value	0.50	0.75	1.00

(c) IWB1 - SNP [A/G] mapped in chromosome 2A

Cluster #	C1	C2	C3
Cluster genotype	AA	AG	GG
Locus 1 (2A)	AA	AG	GG
Locus 2	GG	GG	GG
Locus 3	GG	GG	GG
Theta value	0.67	0.83	1.00

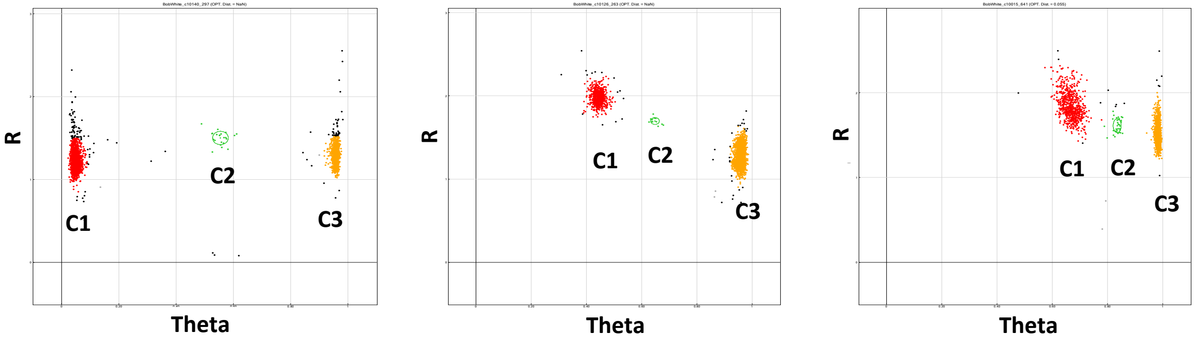

Figure S3. Calling genotypes at the targeted SNP locus from the mean theta value of clusters for assays that detect a (**a**) single-copy, (**b**) duplicated and (**c**) triplicated locus in the wheat genome.
