# Supplementary material for: Characterization of polyploid wheat genomic diversity using a high-density 90 000 single nucleotide polymorphism array
Source: Plant Biotechnol J. 2014 Mar 20;12(6):787–96. doi: 10.1111/pbi.12183 (PMC4265271; doi:10.1111/pbi.12183)
Supplement: Figure S4 — Calling sample genotypes for iSelect assays that detect multiple clusters in a population of unrelated wheat accessions. [file pbi0012-0787-SD7.pdf]

(a) IWB7403 - SNP [A/G] mapped in chromosome 2B

Cluster #	C1	C2	C3
Cluster genotype	AA	GG	GG
Possible locus configuration in wheat genome			
Locus 1 (2B)	AA	GG	GG
Locus 2	AA	AA	AA
Locus 3	AA	AA	--
Theta value	0.00	0.26	0.51

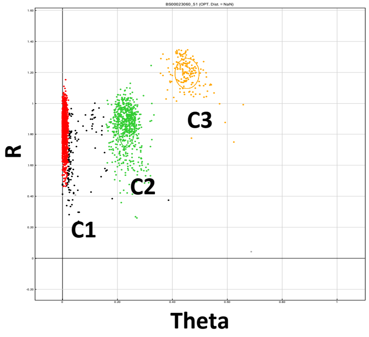

(b) IWB7683 - SNP [A/G] mapped in chromosome 2A

Cluster #	C1	C2	C3
Cluster genotype	AA	GG	GG
Possible locus configuration in wheat genome			
Locus 1 (2A)	AA	GG	GG
Locus 2	AA	AA	--
Theta value	0.00	0.38	0.94

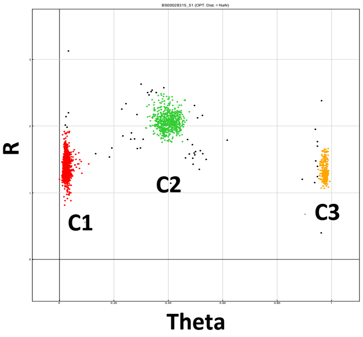

(c) IWB7669 - SNP [A/G] mapped in chromosome 2B

Cluster #	C1	C2	C3
Cluster genotype	AA	Null	GG
Possible locus configuration in wheat genome			
Locus 1 (2B)	AA	--	GG
Theta value	0.00	0.38	0.94

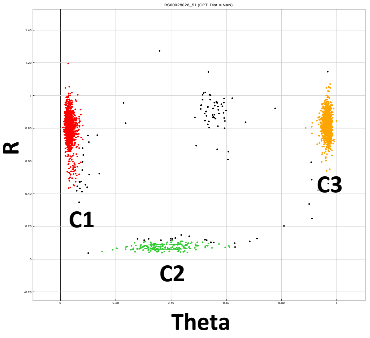

(d) IWB27969 - SNP [T/C] mapped in chromosomes 4A and 4B

Cluster #	C1	C2	C3
Chr. **4A**	TT or tt	tt	TT
Chr. **4B**	TT	CC	CC
Possible locus configuration in wheat genome			
Locus 1 (4A)	TT	tt	TT
Locus 2 (4B)	TT	CC	CC
<u>or</u>			
Locus 1 (4A)	tt	tt	TT
Locus 2 (4B)	TT	CC	CC
Theta value	0.00	0.44	0.59

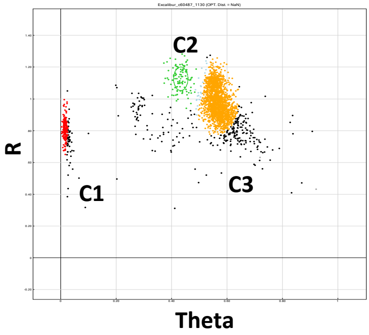

(e) IWB65270 - SNP [A/G] mapped in chromosomes 1B and 2A

Cluster #	C1	C2	C3
Chr. **1B**	AA	AA	GG
Chr. **2A**	AA	GG	GG
Possible locus configuration in wheat genome			
Locus 1 (1B)	AA	GG	GG
Locus 1 (2A)	AA	AA	GG
<u>or</u>			
Locus 1 (1B)	AA	AA	GG
Locus 1 (2A)	AA	GG	GG
Theta value	0.00	0.11	0.32

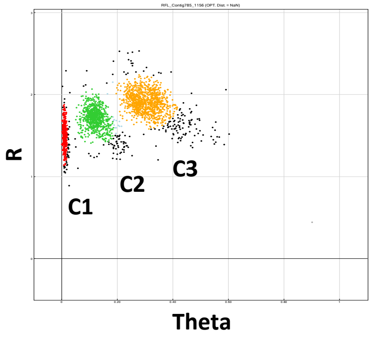

Figure S4. Calling sample genotypes for iSelect assays that detect multiple clusters in a population of unrelated wheat accessions.
