# Supplementary material for: Characterization of polyploid wheat genomic diversity using a high-density 90 000 single nucleotide polymorphism array
Source: Plant Biotechnol J. 2014 Mar 20;12(6):787–96. doi: 10.1111/pbi.12183 (PMC4265271; doi:10.1111/pbi.12183)
Supplement: Figure S5 — LD decay in the populations of wheat cultivars and landraces. [file pbi0012-0787-SD9.pdf]

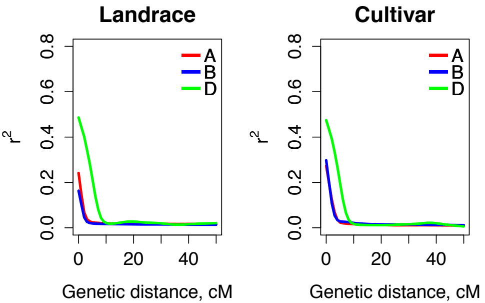

Figure S5. LD decay in the populations of wheat cultivars and landraces. The rate of LD decay was assessed using the SNP markers genetically mapped to the A (red line), B (blue line) and D (green line) genomes. Inter-marker genetic distances were assessed using the consensus genetic map.
