# Supplementary material for: Characterization of polyploid wheat genomic diversity using a high-density 90 000 single nucleotide polymorphism array
Source: Plant Biotechnol J. 2014 Mar 20;12(6):787–96. doi: 10.1111/pbi.12183 (PMC4265271; doi:10.1111/pbi.12183)
Supplement: Table S3 — Durum wheat genotypes used for SNP discovery. [file pbi0012-0787-SD13.docx]

Supplementary Table S3. Durum wheat genotypes used for RNA-seq sequencing and SNP discovery

| Genotype | Pedigree | Genotype | Breeder |
| --- | --- | --- | --- |
| Ciccio^a^ | (Appulo x Valnova) F6 x (Valforte x Patrizio) | Parent of mapping population | Proseme |
| Claudio^b^ | Sel.Cimmyt35/Durango//ISEA1938xGrazia | Parent of mapping population | SIS |
| Kofa^b^ | dicoccum alpha pop-85 S-1 | Parent of mapping population | Westbred |
| Meridiano^b^ | Simeto/WB881//Duilio/F21 | Parent of mapping population | Produttori Sementi S.p.A. |
| Neodur^b^ | 184-7/Valdur//Edmore | Parent of mapping population | Florisem |
| Ofanto^a^ | Adamello x Appulo | Parent of mapping population | Istituto Sperimentale per la Cerealicoltura |
| Pedroso^a^ | n.a. | Parent of mapping population | Semillas Battle |
| Simeto^a^ | Capeiti 8 x Valnova | Parent of mapping population | Proseme |
| Svevo^a^ | Cimmyt line/zenit sib | Parent of mapping population | Produttori Sementi S.p.A. |
| Strongfield^b^ | AC Avonlea’/DT665 | Elite genotype | Agricolture and Agri Food Canada |
| Saragolla^b^ | Iride/O114 | Elite genotype | Produttori Sementi S.p.A. |
| Creso^a^ | CpB 144//Yt54-N10-B/ Cp2 63 Tc | Founder and parent of mapping population | ISEA |
| Altar 84^b^ | RUFF"S"/FG"S"//MEXI75/3/SHWA"S" | Founder | CIMMYT |
| Yavaros 79^b^ | Jori /Anhinga //Flamingo | Founder | CIMMYT |
| Valnova^b^ | Giorgio-324//Senatore Cappelli/Yuma | Founder | Istituto Sperimentale per la Cerealicoltura |
| Cappelli^a^ | Landrace selection from jennah Khetifa | Founder | Istituto Sperimentale per la Cerealicoltura |
| Capeiti 8^b^ | Cappelli/Eiti | Founder | Istituto Sperimentale per la Cerealicoltura |
| Edmore^b^ | D6530//Leeds / Calvin | Founder | Western Plant Breeders |
| Molise Colli^b^ | Selection from *Triticum dicoccum* population | Parent of mapping population | Istituto Sperimentale per la Cerealicoltura |

^a^ Illumina GAIIx analyzer was used to generate 40-76 bp reads; ^b^ HiSeq2000 was used to generate 2 x 100 bp reads.
