# Supplementary material for: Characterization of polyploid wheat genomic diversity using a high-density 90 000 single nucleotide polymorphism array
Source: Plant Biotechnol J. 2014 Mar 20;12(6):787–96. doi: 10.1111/pbi.12183 (PMC4265271; doi:10.1111/pbi.12183)
Supplement: Table S4 — Summary of RNA-seq data generated for cultivar Svevo. [file pbi0012-0787-SD14.docx]

Supplementary Table S4. Summary of RNA-seq data generated for cultivar Svevo

| Tissue/library | Fragment size (bp) | Filtered reads | Sequences (bp) | Avg length (bp) |
| --- | --- | --- | --- | --- |
| Leaf_Fa | 280 | 86,096,832 | 10,193,889,744 | 121.6 |
| Leaf_Fb | 380-480 | 23,756,177 | 2,318,243,566 | 114.7 |
| Root_Ra | 280 | 15,521,963 | 1,667,987,908 | 107.9 |
| Root_Rb | 380-480 | 56,595,216 | 5,576,013,852 | 107.5 |
| Ovaries + anthers at anthesis_Ob | 280 | 33,324,118 | 3,640,576,348 | 102.9 |
| Ovaries + anthers at anthesis_Oa | 380-480 | 76,067,574 | 7,305,100,857 | 103.4 |
| Seed_at milk_stage Gb | 280 | 21,752,415 | 2,374,548,820 | 104.3 |
| Seed_at milk_stage Ga | 380-480 | 57,676,803 | 6,152,936,694 | 106.8 |
| Total |  | >370 M | > 39 Gbp |  |
