# Supplementary material for: Characterization of polyploid wheat genomic diversity using a high-density 90 000 single nucleotide polymorphism array
Source: Plant Biotechnol J. 2014 Mar 20;12(6):787–96. doi: 10.1111/pbi.12183 (PMC4265271; doi:10.1111/pbi.12183)
Supplement: Appendix S1 — Methods (SNP discovery, cluster file development, map construction). [file pbi0012-0787-SD25.docx]

**Supplementary information**

**Identification of SNPs in the transcriptome of tetraploid wheat**

1. The *Triticum turgidum* subsp*. durum* cv. Svevo released in 1996 (CIMMYT line/Zenit) was used to assemble reference transcripts for the durum transcriptome. Total RNA was isolated from multiple tissues using the Total Spectrum Plant RNA (Sigma-Aldrich) according to the manufacturer’s instructions (Table S4). Each library was sequenced on an Illumina GAIIx to generate 150 bp paired-end reads. Following quality trimming and removal of reads originating from cellular organelle, a subset of 66 million reads (~7 Gbp) was randomly selected and used for *de novo* assembly using CLC-Genomics Workbench v4.7 software, which produced a total of 99,885 contigs with a minimum length of 300 bp.
2. Eighteen cultivars, (including Svevo) chosen from a worldwide *T. durum* elite germplasm collection used for association mapping (Table S3) (Maccaferri et al., 2011), and one Italian selection of emmer wheat (*T. dicoccum*), were used to identify transcriptome-derived SNPs. cDNA libraries for each genotype were prepared for sequencing from both coleoptiles and young leaves of two-week-old seedlings using the Illumina TruSeq RNA kit according to the manufacturer’s protocol.
3. In the first experiment, cDNA libraries were sequenced using the Illumina Genome Analyzer IIx to generate 40 - 76 bp reads (Table S4). Reads were aligned to the hexaploid wheat (*T. aestivum*) NCBI unigene set of 40,349 genes (28.5 Mbp). In the second experiment, cultivars were sequenced using the HiSeq2000 (2 x 100 bp reads). Quality trimmed reads were aligned to the Svevo RTs using CLC-Genomics Workbench v4.7 (window length =11, maximum gap and mismatch count = 6, minimum central quality = 20, minimum average quality = 15, minimum coverage = 8, maximum coverage = 1,000,000, sufficient variant count threshold = 1000 and required variant count threshold = 4). Custom scripts were used to identify SNPs. A SNP was declared when at least 5 varieties had a minimum read coverage of 8 and at least 3 varieties showed variation compared to the reference.

**Identification of SNPs in the transcriptome of hexaploid wheat**

1. SNP discovery was performed in the transcriptomes of 19 accessions of hexaploid wheat (Table S1) sequenced using Illumina (GAII_x_ and HiSeq2000) next-generation sequencing platforms (Cavanagh et al., 2013). Barcoded samples were pooled and sequenced on a single lane of HiSeq2000 instrument (2 x 100 bp reads). Read quality trimming was performed using the fastx-toolkit (hannonlab.cshl.edu/fastx_toolkit/‎) using the following parameters: 1) fastq_quality_trim -t 20 -l 30 and 2) fastq_quality_filter -q 20 -p 80. We generated a reference transcriptome (RT) by combining full-length cDNA sequences available at the TriFLDB database (Mochida et al., 2009) with previously generated homoeolog-specific transcript assemblies based on 454 sequencing of wheat transcriptome (Akhunov et al., 2013; Cavanagh et al., 2013). The major drawback of SNP mining in a polyploid species is the elevated false SNP call rate resulting from duplicated homoeologous regions in the wheat genome. Because of high variability in the level of divergence between the wheat homoeologous genomes (Akhunov et al., 2013), it is difficult to select a single set of read mapping parameters, which would allow all reads to map “uniquely”. Therefore, we selected low stringency mapping parameters that allowed for reads from the duplicated genes to map to the diverged copies of the homoeologous or paralogous RTs. The following parameters were used for read mapping with the *bowtie* program (Langmead et al., 2009): -a -n 3 -e 200 -p 30.

The SAMtools program was used to discover variable sites with a minimum coverage depth of 4. The resulting raw variant calls included both true SNPs and homoeolog-specific variable (HSV) sites. Variable sites showing more than two alleles were discarded. A custom Perl script was used to parse the SAM files and calculate the read coverage depth at variable sites. The following rules for genotype calling were applied: 1) the genotype was called AB if a variable site had two alleles (A and B) and the coverage depth of each allele was ≥2; 2) the genotype was called A (or B) if the variable site had only one allele with the coverage depth ≥8; 3) in the remaining cases the genotype call was recorded as missing. The presence of both variants in all analyzed accessions (AB genotype calls) is a characteristic feature of homoeologous variable sites (HSV) that was used as a criterion for their filtering. For true SNPs, we expected to see both AB and A (or B) genotype calls among accessions. We further filtered for SNPs that showed high frequency in the discovery panel by selecting sites for which at least 4 accessions had genotype data and at least two accessions show alternative genotypes. To validate SNPs, a set of 100 randomly chosen polymorphic sites between the Kukri and RAC875 cultivars was re-sequenced by the Sanger approach.

Re-sequencing of sites polymorphic between accessions Kukri and RAC875 validated about 73% of SNPs (53 out of 73) (Table S2), comparable to the validation rates obtained for polyploid crops in other studies (Allen et al., 2011; Cavanagh et al., 2013; Edwards et al., 2012; Lai et al., 2012). We did not observe an effect of low coverage, low MAF or repetitive DNA on the rate of SNP prediction. SNP calling in the wheat genome is quite complicated due to polyploidy, a high rate of inter- and intra-chromosomal segment duplication (~25%) (Akhunov et al., 2003) and high repetitive DNA content. We found that the rate of our SNP validation was comparable to other studies. In a study reporting the development of the 9K wheat iSelect assay, the validation rate was 85% (Cavanagh et al. 2013). In other studies, where SNPs were discovered by wheat transcriptome sequencing, the rate of SNP validation was 67% (Allen et al., 2011). Low-coverage whole genome sequencing resulted in SNP calls showing about 90% concordance with the SNPs used to design the 9K iSelect assay. If we will take into account the validation rate for 9K iSelect assay (85%) (Cavanagh et al., 2013), then the expected validation for this dataset will be 0.85 x 0.90 x 100% = 77%, which is only slightly higher than our experimental estimates.

***In silico* assignment of SNPs to the wheat chromosomes**

A two-step approach was used to assign the 91,829 SNPs on the Infinium iSelect array to wheat chromosomes. First, potential hybridization sites in the wheat genome for the Infinium assay SNP detection probes were identified. The 50-bp nucleotide sequences corresponding to the SNP detection probes on the Infinium iSelect array were used as blastn queries against the assembled chromosome survey sequence (CSS) contigs of flow-sorted wheat chromosomes from variety Chinese Spring (International Wheat Genome Sequencing Consortium, http://wheat-urgi.versailles.inra.fr/Seq-Repository/). A SNP detection probe was assumed to hybridize to a wheat locus when sequence identity exceeded 80% and there were no more than 2 mismatches within the first ten nucleotides from the 3’ end of the detection probe. Custom perl scripts were used to extract the position of nucleotides interrogated by the SNP detection probes within CSS contigs.

The hybridization site at which the target SNP was located was determined using a subset of varieties from the SNP discovery panel. This was achieved by aligning transcriptome and whole genome shotgun sequence generated using 100-bp paired end sequencing for nine wheat varieties (Cavanagh et al., 2013; Edwards et al., 2012) to the CSS contigs using BWA (Li and Durbin, 2009) with default settings. SAMtools (Li et al., 2009) was used to extract from the resulting alignment files the nucleotide present in each wheat variety at the interrogated positions on all GSS contigs. When polymorphism for the targeted SNP was not observed among the nine varieties, the chromosomal location for the targeted SNPs was inferred from the best BlastN hit (based on percent identity) for alignment of sequences flanking the SNP against the CSS contigs. The cumulative number of 90K iSelect probes’ hybridization sites in the wheat genome is presented in Figure S1.

**SNP genotype calling of bi-parental mapping crosses using the polyploid version of Genome Studio**

The following three step process was used to for genotype calling in bi-parental doubled haploid and recombinant inbred line mapping populations:

***Step 1***

1. Set DBSCAN and OPTICS clustering algorithm parameters *Cluster Distance* to 0.07 and *Minimum Number of Points in Cluster* to 10 by clicking on **Tools** in the main menu bar and selecting **Current Project Options**.
2. Select all SNPs in **SNP Table**, right click over the selected SNPs and choose *Cluster Selected SNPs* in the menu that appears, then select *DBSCAN* in the sub-menu.
3. Sort **SNP Table** by *# Clusters* and *Call Freq* in ascending order.
4. Select SNPs with *# Clusters* equal to 2 and *Call Freq* >0.9, and set *Aux* to 1.
5. Sort **SNP Table** by *Aux* in descending order then by *C1 Freq* and *C2 Freq* in ascending order.
6. Select SNPs with *C1 Freq* >0.35 and *C1 Freq* <0.65, and set *Comment* as “Polymorphic_Step1”.
7. Select all SNPs and set *Aux* value to 0.
8. Select SNPs with *# Clusters* greater than 2, and set *Comment* as “Multiple Clusters”.

***Step 2***

1. Select SNPs with *# Clusters* equal to 1 in **SNP Table**, right click over the selected SNPs, choose *Cluster Selected SNPs* in the menu that appears, then select *2 Clusters* in the sub-menu.
2. Sort **SNP Table** by *Comment* in descending order, *# Clusters* and *Call Freq* in ascending order.
3. Select SNPs with *# Clusters* equal to 2 and *Call Freq* >0.9, and set *Aux* value to 1.
4. Sort **SNP Table** by *Aux* in descending order, then by *C1 Freq* and *C2 Freq* in ascending order.
5. Select SNPs with *C1 Freq* >0.35 and *C1 Freq* <0.65, and set *Comment* as “Polymorphic_Step2”.
6. Select all SNPs and set *Aux* value to 0.
7. Sort **SNP Table** by *Comment* in descending order, then by *# Clusters* and *Call Freq* in ascending order.
8. Select SNPs with *# Clusters* equal to 1, and set *Comment* as “Monomorphic”.
9. Select SNPs with *# Clusters* equal to 2 and *Call Freq* <0.2, and set *Comment* as “Monomorphic”.

***Step 3***

1. Set DBSCAN clustering algorithm parameter *Cluster Distance* to 0.09. Increase the cluster distance allows the identification of clusters that were too broad to be detected in the first step.
2. Select SNPs in **SNP Table** that do not have an annotation in *Comment*, right click over the selected SNPs and choose *Cluster Selected SNPs* in the menu that appears, then select *DBScan* in the sub-menu.
3. Sort **SNP Table** by *Comment* in descending order, then by *Custum Cluster #* and *Call Freq* in ascending order.
4. Select SNPs with *# Clusters* equal to 2 and *Call Freq* > 0.9, and set *Aux* value to 1.
5. Sort **SNP Table** by *Aux* in descending order, then by *C1 Freq* and *C2 Freq* in ascending order.
6. Select SNPs with *C1 Freq* >0.35 and *C1 Freq* <0.65, and set *Comment* as “Polymorphic_Step3”.
7. Select all SNPs and set *Aux* value to 0.
8. Select SNPs with *# Clusters* >2, and set *Comment* as “Multiple Clusters”.
9. Select SNPs with *# Clusters* equal to 1 and *Call Freq* >0.99, and set *Comment* as “Monomorphic”.
10. Finally, sort **SNP Table** by *Comment* and visually check clustering for SNPs marked as “Multiple Clusters”. Manually curate incorrectly clustered SNPs.

To assess the consistency of the described genotype calling procedure, we performed genotype calling in different laboratories in USA (E. Akhunov) and Australia (M. Hayden). The SynOp mapping population dataset was used for this purpose. Comparison of genotyping data generated for 150 recombinant inbred lines in each laboratory showed 99.8% concordance suggesting high reproducibility of the developed genotype calling method.

**Cluster file development for unrelated accessions using the polyploid version of Genome Studio**

Several strategies were investigated to streamline cluster file development for genotype calling in the worldwide collection of 2,500 unrelated hexaploid wheat accessions. While the strategy for SNP genotype calling in bi-parental mapping crosses worked well for assays that revealed biallelic inheritance, or had multiple well-spaced clusters due to segregation at more than one duplicated locus, manual checking of individual assays was required to ensure correct clustering. The requirement for manual checking was exasperated by the absence of *a priori* knowledge for expected allele frequencies in the unrelated population of wheat accessions.

The most efficient strategy identified for cluster file development was based on sequential addition of bi-parental mapping populations followed by detection of new clusters after each step. Briefly, the approach starts with cluster detection in the first bi-parental population (as described in *SNP genotype calling of bi-parental mapping crosses using polyploid version of Genome Studio*) and generation of a cluster (egtp) file. The egpt file was then applied (with parameter *Cluster Distance Limit* set at 3 standard deviations) to a new project that contained both the first and second bi-parental mapping populations. The **SNP Table** was sorted by *Call Freq* and SNPs with less than 90% call frequency were selected. The egtp file was reapplied (with parameter *Cluster Distance Limit* set at 4 standard deviations) to the selected SNPs. This step allowed better capture of sample data points in broad clusters. The **SNP Table** was again sorted by *Call Freq* and SNPs with less than 80% call frequency were selected and re-clustered using the approach described for SNP genotype calling in bi-parental mapping populations. Following completion of this cluster recognition training, a new cluster egtp file was generated after applying a *Confidence Cut-off Score* of 0.8 (which is a measure of confidence that a given sample belongs to a specified cluster, as opposed to any other cluster), and applied to a new GenomeStudio project containing the first, second and third mapping populations. This procedure was repeated for each mapping population.

The efficiency of the sequential cluster file development approach is illustrated in Table S9. The two different cluster files developed using two bi-parental crosses, correctly captured polymorphism for 20.9% (on average) of the functional assays. After sequential addition of the same two mapping populations, polymorphism was correctly captured for 34.8% of the functional assays. Inclusion of a third mapping population increased the proportion of assays capturing polymorphism to 46.1%. The success of the sequential approach was due to the detection of new polymorphisms by inclusion of both populations in the same GenomeStudio project; for example when the populations were fixed for alternate SNP alleles (Figure S2a). The sequential approach also simplified cluster file development since the expected allele frequency was known, which facilitated efficient filtering of the SNPs using the process described for genotype calling of bi-parental mapping crosses (Figure S2b, c). Sequential addition of six bi-parental DH mapping populations resulted in 48,316 (59.2%) of the 81,587 functional assays being trained to capture polymorphism, which amounted to 85.7% (48,316/56,388) of the functional assays that visually revealed polymorphism.

**Conversion of cluster assignments to genotype calls in polyploid version of GenomeStudio**

The genotype of a sample for the SNP locus specifically targeted in an iSelect assay can be determined from the mean theta value of the clusters. Similarly, the position of clusters can be used to infer arbitrary genotypes for non-targeted polymorphism such as uncharacterized sequence variation that occurs within the annealing site of the oligonucleotide probe. The level of confidence for which a genotype can be assigned depends on the complexity of the clustering pattern. Confidence is highest when the allelic relationship between clusters has been previously established, for example by genetic mapping.

For Infinium type II assays, a single oligonucleotide probe (designed to anneal adjacent to the targeted SNP) is used to interrogate the nucleotide at the SNP position in a single base extension reaction using fluorescently labeled nucleotides, where adenine and thymine are labeled with Cy5 and cytosine and guanine are labeled with Cy3. The SNP genotype for a sample is derived from the ratio of fluorescence for the incorporated nucleotides, which is calculated as theta = 2/π tan^–1^(Cy5/Cy3). A theta value of 0 represents pure Cy5 signal (A or T allele) and 1 represents pure Cy3 signal (C or G allele). The amount of fluorescent signal generated for a sample is represented by normR, which is the normalized intensity for the sample across all samples. When duplicate (homoeologous and paralogous) copies of the SNP locus are present in the genome, theta and normR represent aggregate scores for the total fluorescence signal generated by the simultaneous assay of each duplicated copy of the locus (Table S10).

Figure S3 illustrates the assignment of sample genotypes at the targeted SNP locus for representative iSelect assays that show three distinct clusters corresponding to the AA, AB and BB genotypes expected for a biallelic SNP segregating in the population of 2,500 unrelated worldwide accessions. Sample genotypes are directly inferred from the mean theta values of the clusters.

Figure S4 illustrates sample genotype assignment for iSelect assays in which multiple clusters were observed in the population of 2,500 worldwide accessions. In these representative assays, three major clusters corresponding to homozygous genotypes were observed. Clusters corresponding to heterozygous genotypes are not shown for simplicity, but are expected to occur about half way between allelic clusters corresponding to the reference and alternate homozygous states.

The three major clusters revealed by assay IWB7403 (Figure S4a) were shown to genetically map to a single locus in chromosome 2B. Clusters C1 and C2 segregated in the AUS/Bts and Sun/AUS populations, while clusters C1 and C3 segregated in the AUS/Yo cross. Based on the mean theta values for the cluster positions, the genotype at the mapped SNP locus is AA for cluster C1, and GG for clusters C2 and C3. While it cannot be conclusively inferred, the theta shift observed for clusters C2 and C3 might be due to the assay of only two duplicated copies of the SNP locus in samples assigned to cluster C3 and three duplicated copies of the locus in samples assigned to cluster C2 (Table S10). Irrespective of the cause for the observed theta shift between clusters C2 and C3, sample genotype assignment for the targeted SNP position in chromosome 2B can be assigned with high confidence for all three clusters.

Similar reasoning can be used to infer sample genotypes for the three major clusters revealed by assay IWB7683 (Figure S4b). Genetic mapping in the AUS/Bts and Cha/Glen populations showed that clusters C1 and C3 were allelic and to mapped to chromosome 2A. In the AUS/Yo population, clusters C1 and C2 were allelic and mapped the same locus in chromosome 2A. Based on mean theta values for cluster positions, the genotype at the mapped SNP locus is AA for cluster C1, and GG for clusters C2 and C3. Similarly, while it cannot be conclusively inferred the theta difference for clusters C2 and C3 is consistent with the assay of two duplicated copies of the SNP locus in samples assigned to cluster C2, and detection of only the chromosome 2A locus in samples assigned to cluster C3 (Table S10). Again, sample genotype assignment for the targeted SNP position in chromosome 2A can be assigned with high confidence.

The three major clusters revealed by assay IWB7669 (Figure S4c) were shown to genetically map to a single locus on chromosome 2B. Clusters C1 and C2 segregated in the AUS/Yo population, while clusters C1 and C3 segregated in the AUS/Bts, Cha/Glen, Op/Syn and Sun/AUS crosses. Based on mean theta values for the clusters, the sample genotypes at the mapped SNP locus are AA and GG for clusters C1 and C3, respectively. The genotype for samples assigned to cluster C2 can only be implied since the sequence variation responsible for the observed polymorphism is unknown. The low normR value for the cluster position is consistent with failure of oligonucleotide probe annealing, which could be due either to presence-absence variation for this single copy SNP locus in the wheat genome, or uncharacterized mutations within the oligonucleotide probe annealing site that prevent probe hybridization or single base extension. Given the low normR value for the cluster, the genotype for cluster C2 can be arbitrarily denoted as a null allele. Hence, three alleles at the chromosome 2B locus can be confidently assigned to samples: AA, GG and null/null for clusters C1, C3 and C2, respectively.

The three clusters revealed by assay IWB27969 (Figure S4d) were shown to genetically map to homoeoloci in chromosomes 4A and 4B. In the AUS/Bts and AUS/Yo populations clusters C1 and C3 mapped in chromosome 4B, while in the Cha/Glen and Sun/AUS crosses clusters C2 and C3 mapped in chromosome 4A. Based on mean theta values for cluster positions (which suggest a total of three duplicated copies of the SNP locus were assayed), it is expected that the genomic location of the targeted SNP was chromosome 4B, as the difference in theta value between clusters C1 and C3 closely followed theoretical expectations for a hexaploid dose locus located on each of three homoeologous group 4 chromosomes (Table S10). Further supporting this assumption was the assignment of the targeted SNP to chromosome 4B using transcriptome and whole genome shotgun sequence available for nine wheat varieties from the discovery panel (Table S8). Based on this assumption, the sample genotype at the mapped chromosome 4B locus segregating in AUS/Bts and AUS/Yo is TT and CC for clusters C1 and C3, respectively. In contrast, sample genotypes for clusters C2 and C3 segregating in the Cha/Glen and Sun/AUS crosses cannot be directly inferred. The difference in theta value between clusters C2 and C3 does not follow theoretical expectations for segregation of polymorphism at the nucleotide position interrogated by the single base extension chemistry; i.e. the observed theta difference of 0.15 is less than the theoretical value of 0.33 and 0.50 expected for segregation at an interrogated SNP position for a hexaploid and tetraploid dose locus, respectively (Table S10). Rather, the theta difference is more suggestive for segregation at the mapped chromosome 4A locus of an uncharacterized mutation within the oligonucleotide probe annealing site that reduces assay detection sensitivity in samples assigned to cluster C2, compared to samples assigned to cluster C3. Based on mean theta values, both clusters C2 and C3 have a cytosine nucleotide at the SNP position interrogated by single base extension. As the nucleotide variation responsible for the observed polymorphism at the mapped chromosome 4A locus cannot be directly inferred, the arbitrary genotype *cc* is assigned to cluster C2 and CC to cluster C3. Despite this complexity, it is still possible to confidently assign sample genotypes at the two mapped loci in the population of 2,500 unrelated individuals. Samples assigned to cluster C1 have genotypes TT/TT, samples assigned to cluster C2 have genotypes *cc*/CC, and those assigned to cluster C3 have genotypes CC/CC at the chromosome 4A and 4B loci, respectively.

In contrast, sample genotypes for the major clusters revealed by assay IWB65270 (Figure S4e) can only be confidently assigned for clusters C1 and C3 in the population of 2,500 unrelated accessions. In the Sundor/AUS cross, clusters C2 and C3 mapped to chromosome 1B, whereas in the Wes/Kauz population clusters C1 and C2 mapped to chromosome 2A. Based on mean theta values for cluster positions (which suggest a total of six duplicated copies of the SNP locus were assayed), the genotype at the mapped chromosome 1B locus in the Sun/AUS population is AA for cluster C2 and GG for cluster C3, while at the mapped chromosome 2A locus in the Wes/Kauz cross the samples genotypes for clusters C1 and C2 is AA and GG, respectively. While unambiguous sample genotype assignment is possible within the bi-parental mapping populations, overlap of cluster positions prevents accurate genotype assignment for samples in cluster C2 in the populations of 2,500 unrelated accessions. Assay IWB65270 highlights the possibility for different allelic configurations to generate similar (or even the same) theta values. This is expected, since the fluorescence signal observed for a sample is the sum of all loci assayed. Assay IWB65270 demonstrates the value of *a priori* knowledge for the allelic relationship between clusters for confidently assigning genotypes to samples in unrelated populations.

**Construction of individual and consensus genetic maps**

To construct the genetic maps, SNPs with more than 30% missing data were removed. Preliminary linkage maps were constructed using the MSTmap program (Wu et al., 2008) with the Kosambi distance function and p-value cutoff of 10^-6^ for clustering markers to linkage groups (LGs). The resulting LGs were assigned to individual chromosomes based on the best blastn hit from a comparison of SNP-flanking sequences with the sequences of flow-sorted wheat chromosomes. LGs assigned to the same chromosome based on blastn analysis were re-analyzed using MSTmap with a LG clustering cut off p-value of <0.01. Next, each population was individually processed using R/qtl (Arends, Prins, Jansen, & Broman, 2010) to remove individuals with >99% genetic similarity, switch parental alleles for markers displaying high recombination fractions and high LOD scores, and merge linkage groups based on the re-estimated recombination fractions. Markers having zero recombination fraction within a LG were binned together and reordered using R/mpmap (B Emma Huang & George, 2011). Map distances were estimated for the binned map, and markers within a bin were positioned at the bin location to produce the final genetic maps.

A total of 45,109 assays revealed polymorphism in the mapping populations (Tables S12, S13). Of these assays, 44,345 could be mapped to one or more of 46,977 loci on specific wheat chromosomes. Of the remaining 764 polymorphic assays, 20 mapped to linkage groups that could not be unambiguously assigned to a wheat chromosome, and 744 were not linked with any other markers. No unusual clustering patterns were reported for these 744 SNPs, which showed expected segregation ratio (1:1) in the mapping populations. It is likely that these 744 assays either identify unlinked polymorphisms resulting from the use of synthetic parents in four of the bi-parental mapping populations or represent genotyping errors. However, even if all these unmapped 744 SNPs are due to genotyping errors, the error rate is low (1.6%) and could be associated with semi-automated clustering and genotype calling applied to a large dataset.

The consensus map was constructed using six bi-parental doubled haploid mapping populations (BT-Schomburgk x AUS33384, Young x AUS33414, Chara x Glenlea, W7984 × Opata M85, Sundor x AUS30604 and Westonia x Kauz) since these were the only populations available at the time. The consensus map was constructed using MergeMap (Wu, Close, & Lonardi, 2011) to convert each linkage map into directed acyclic graphs that were merged into a consensus based on their shared vertices. Initially, equal weights (1.0) were assigned to each chromosome for all LGs specified in the MergeMap configuration file. Due to the presence of the alien *Sr36* introgression in cultivar Young and knowledge that its presence restricts recombination and complicates map construction (Bevan E Huang et al., 2012), the linkage group for chromosome 2B in the Young x AUS33414 cross was excluded from the consensus build. Based on marker number, recombination and conflicts identified by MapMerge for each chromosome, weights were assigned to individual groups and the consensus map was recalculated. Individual maps were then visually checked, and any erroneous linked groups were removed before repeating MergeMap analysis.

In bi-parental mapping populations we found an increase (about 30%) in the length of genetic maps compared to maps constructed using low-density genotyping data. The inflated genetic distances between the markers are unlikely caused by high genotype calling error rates. They are due to the accumulation of low levels of genotyping error in maps, which now currently harbour about 10X more markers than genetic maps previously reported. Similar inflation of genetic distances on a high-density consensus genetic map was previously described for barley (Muñoz-Amatriaín et al., 2011). The genetic distances on the consensus map have been scaled using the SynOp DH genetic map as previously described (Cavanagh et al., 2013).

**Linkage disequilibrium analysis**

Pair-wise linkage disequilibrium (LD) was measured as squared allele-frequency correlations, *r*^2^. To reduce the variation of LD estimates only SNP alleles with minor allele frequency (MAF) > 0.05 were used. The rate of LD decay was assessed by plotting *r*^2^ for SNP pairs against genetic distance. The trends in LD decay were summarized by fitting a locally-weighted linear regression (loess) line. The LD estimates were obtained for the population of 127 landraces and 423 wheat cultivars (Fig. S5). LD in the A and B genomes of cultivars and landraces decayed within 3 cM and 5 cM (*r*^2^ < 0.05), respectively; in the D genome LD decayed within 9-10 cM (Fig. S5).

**Supplementary References:**

Akhunov, E. D., Goodyear, A. W., Geng, S., Qi, L., Echalier, B., Gill, B. S., … Dvorak, J. (2003). The Organization and Rate of Evolution of Wheat Genomes Are Correlated With Recombination Rates Along Chromosome Arms. *Genome Research*, *13*, 753–763. doi:10.1101/gr.808603.

Akhunov, E. D., Sehgal, S., Liang, H., Wang, S., Akhunova, A. R., Kaur, G., … Gill, B. S. (2013). Comparative analysis of syntenic genes in grass genomes reveals accelerated rates of gene structure and coding sequence evolution in polyploid wheat. *Plant physiology*, *161*(1), 252–65. doi:10.1104/pp.112.205161

Allen, A. M., Barker, G. L. A., Berry, S. T., Coghill, J. A., Gwilliam, R., Kirby, S., … Edwards, K. J. (2011). Transcript-specific, single-nucleotide polymorphism discovery and linkage analysis in hexaploid bread wheat (Triticum aestivum L.). *Plant biotechnology journal*, *9*(9), 1086–99. doi:10.1111/j.1467-7652.2011.00628.x

Arends, D., Prins, P., Jansen, R. C., & Broman, K. W. (2010). R/qtl: high-throughput multiple QTL mapping. *Bioinformatics (Oxford, England)*, *26*(23), 2990–2. doi:10.1093/bioinformatics/btq565

Cavanagh, C. R., Chao, S., Wang, S., Huang, B. E., Stephen, S., Kiani, S., … Akhunov, E. (2013). Genome-wide comparative diversity uncovers multiple targets of selection for improvement in hexaploid wheat landraces and cultivars. *Proceedings of the National Academy of Sciences of the United States of America*, *110*(20), 8057–62. doi:10.1073/pnas.1217133110

Edwards, D., Wilcox, S., Barrero, R. a, Fleury, D., Cavanagh, C. R., Forrest, K. L., … Fitzgerald, A. (2012). Bread matters: a national initiative to profile the genetic diversity of Australian wheat. *Plant biotechnology journal*, *10*(6), 703–8. doi:10.1111/j.1467-7652.2012.00717.x

Huang, B Emma, & George, A. W. (2011). R/mpMap: a computational platform for the genetic analysis of multiparent recombinant inbred lines. *Bioinformatics (Oxford, England)*, *27*(5), 727–9. doi:10.1093/bioinformatics/btq719

Huang, Bevan E, George, A. W., Forrest, K. L., Kilian, A., Hayden, M. J., Morell, M. K., & Cavanagh, C. R. (2012). A multiparent advanced generation inter-cross population for genetic analysis in wheat. *Plant biotechnology journal*, *10*(7), 826–39. doi:10.1111/j.1467-7652.2012.00702.x

Lai, K., Duran, C., Berkman, P. J., Lorenc, M. T., Stiller, J., Manoli, S., … Edwards, D. (2012). Single nucleotide polymorphism discovery from wheat next-generation sequence data. *Plant biotechnology journal*, *10*(6), 743–9. doi:10.1111/j.1467-7652.2012.00718.x

Langmead, B., Trapnell, C., Pop, M., & Salzberg, S. L. (2009). Ultrafast and memory-efficient alignment of short DNA sequences to the human genome. *Genome biology*, *10*(3), R25. doi:10.1186/gb-2009-10-3-r25

Li, H., & Durbin, R. (2009). Fast and accurate short read alignment with Burrows-Wheeler transform. *Bioinformatics (Oxford, England)*, *25*(14), 1754–60. doi:10.1093/bioinformatics/btp324

Li, H., Handsaker, B., Wysoker, A., Fennell, T., Ruan, J., Homer, N., … Durbin, R. (2009). The Sequence Alignment/Map format and SAMtools. *Bioinformatics (Oxford, England)*, *25*(16), 2078–9. doi:10.1093/bioinformatics/btp352

Maccaferri, M., Sanguineti, M. C., Demontis, A., El-Ahmed, A., Garcia del Moral, L., Maalouf, F., … Tuberosa, R. (2011). Association mapping in durum wheat grown across a broad range of water regimes. *Journal of experimental botany*, *62*(2), 409–38. doi:10.1093/jxb/erq287

Mochida, K., Yoshida, T., Sakurai, T., Ogihara, Y., & Shinozaki, K. (2009). TriFLDB: a database of clustered full-length coding sequences from Triticeae with applications to comparative grass genomics. *Plant physiology*, *150*(3), 1135–46. doi:10.1104/pp.109.138214

Muñoz-Amatriaín, M., Moscou, M. J., Bhat, P. R., Svensson, J. T., Bartoš, J., Suchánková, P., … Close*, T. J. (2011). An Improved Consensus Linkage Map of Barley Based on Flow-Sorted Chromosomes and Single Nucleotide Polymorphism Markers. *The Plant Genome Journal*, *4*(3), 238. doi:10.3835/plantgenome2011.08.0023

Wu, Y., Bhat, P. R., Close, T. J., & Lonardi, S. (2008). Efficient and accurate construction of genetic linkage maps from the minimum spanning tree of a graph. *PLoS genetics*, *4*(10), e1000212. doi:10.1371/journal.pgen.1000212

Wu, Y., Close, T. J., & Lonardi, S. (2011). Accurate construction of consensus genetic maps via integer linear programming. *IEEE/ACM transactions on computational biology and bioinformatics / IEEE, ACM*, *8*(2), 381–94. doi:10.1109/TCBB.2010.35
